# Heterokaryon Incompatibility Is Suppressed Following Conidial Anastomosis Tube Fusion in a Fungal Plant Pathogen

**DOI:** 10.1371/journal.pone.0031175

**Published:** 2012-02-02

**Authors:** Francine H. Ishikawa, Elaine A. Souza, Jun-ya Shoji, Lanelle Connolly, Michael Freitag, Nick D. Read, M. Gabriela Roca

**Affiliations:** 1 Departamento de Biologia, Universidade Federal de Lavras, Lavras, Minas Gerais, Brazil; 2 Department of Biochemistry and Biophysics, Center for Genome Research and Biocomputing, Oregon State University, Corvallis, Oregon, United States of America; 3 Fungal Cell Biology Group, Institute of Cell Biology, University of Edinburgh, Edinburgh, United Kingdom; University of Minnesota, United States of America

## Abstract

It has been hypothesized that horizontal gene/chromosome transfer and parasexual recombination following hyphal fusion between different strains may contribute to the emergence of wide genetic variability in plant pathogenic and other fungi. However, the significance of vegetative (heterokaryon) incompatibility responses, which commonly result in cell death, in preventing these processes is not known. In this study, we have assessed this issue following different types of hyphal fusion during colony initiation and in the mature colony. We used vegetatively compatible and incompatible strains of the common bean pathogen *Colletotrichum lindemuthianum* in which nuclei were labelled with either a green or red fluorescent protein in order to microscopically monitor the fates of nuclei and heterokaryotic cells following hyphal fusion. As opposed to fusion of hyphae in mature colonies that resulted in cell death within 3 h, fusions by conidial anastomosis tubes (CAT) between two incompatible strains during colony initiation did not induce the vegetative incompatibility response. Instead, fused conidia and germlings survived and formed heterokaryotic colonies that in turn produced uninucleate conidia that germinated to form colonies with phenotypic features different to those of either parental strain. Our results demonstrate that the vegetative incompatibility response is suppressed during colony initiation in *C. lindemuthianum*. Thus, CAT fusion may allow asexual fungi to increase their genetic diversity, and to acquire new pathogenic traits.

## Introduction

Fungal plant pathogens exhibit a high degree of genetic variability but how this is acquired in the apparent absence of sexual reproduction is not well understood. *Colletotrichum lindemuthianum* is an important pathogenic fungus that causes anthracnose on *Phaseolus vulgaris*, the common bean. Although effective control of this disease is an urgent issue for improving bean cultivation, this has proven difficult due to the large degree of pathogenic, and presumably genetic, variation observed between different *C. lindemuthianum* strains [1], [2], [3]. Generation of high genetic variability may involve genetic recombination between different isolates but how exactly this is achieved remains unclear as sexual reproduction in *C. lindemuthianum*, which facilitates meiotic genetic recombination, is rarely observed in nature [4], [5].

One possible source of new genetic variability is vegetative hyphal fusion allowing horizontal gene or chromosome transfer that may be followed by parasexual (i.e. non-meiotic) recombination [6], [7]. It is becoming clear that horizontal gene and chromosome transfer can have an important impact on the emergence of fungal pathogens adapted to new hosts. However, the significance of heterokaryon incompatibility responses (often termed vegetative incompatibility reactions) in preventing successful vegetative hyphal fusion in nature is little understood [8], [9], [10], [11].

Non-self fusion between mature hyphae of genetically distinct filamentous fungi results in the formation of heterokaryotic cells in which nuclei with different genotypes share the same cytoplasm. However, if the fused cells have different alleles at their heterokaryon or vegetative incompatibility (*het* or *vic*) loci then they are vegetatively incompatible and the fusion commonly results in rapid death of the heterokaryotic cell formed [10], [12]. This vegetative incompatibility response has been interpreted as a defence mechanism to prevent transmission of undesirable genetic elements, such as viruses, or mitochondria [13]. Thus, although the development of vegetative heterokaryotic cells may be advantageous for filamentous fungi, the genetic mechanism of vegetative incompatibility may also restrict the process between two genetically different individuals. It has recently been shown that genetic diversity in natural populations of *C. lindemuthianum* is considerable and that numerous vegetative compatibility groups are present [14]. However, our knowledge of vegetative incompatibility in *Colletotrichum* species is still very limited [7], [14]. In particular, it is not known whether hyphal fusion can lead to the formation of stable heterokaryotic cells that may result in parasexual recombination, which in turn may account for the high degree of genetic variability in these plant pathogens.

The conidial anastomosis tube (CAT), a specialized hypha or cell protrusion involved in somatic cell fusion during colony initiation, was first described in *C. lindemuthianum* [15]. CATs have since been shown to be commonly formed by filamentous fungi, including many plant pathogens [16]. They are distinct from the ‘fusion hyphae’ that are formed in mature colonies, and allow the formation of interconnected networks of conidia and conidial germlings [17], [18], [19]. Heterokaryon formation between compatible strains of *C. lindemuthianum* was first described in fused conidia linked by CATs [15]. Evidence has been obtained that CAT fusion can occur between the two species, *C. lindemuthianum* and *C. gossypii*, and that the heterokaryotic cells believed to have been formed possessed phenotypic traits of these two vegetatively incompatible species [20]. Horizontal chromosome transfer has been demonstrated between two vegetatively incompatible biotypes of *C. gloeosporioides* co-cultured under laboratory conditions and this is also thought to occur in nature [21], [22]. It has been suggested that slow-growing heterokaryons formed between incompatible biotypes of *C. gloeosporoides* may represent intermediates in supernumerary chromosome transfer resulting from CAT fusion [23].

In order to follow up on earlier observations of heterokaryon formation in *C. lindemuthianum*, we generated strains in which nuclei were labelled with either a green fluorescent protein (GFP) or red fluorescent protein (RFP). The dynamics and fates of the different types of labelled nuclei from vegetatively incompatible strains of *C. lindemuthianum* were monitored following vegetative hyphal fusion between mature colonies and CAT fusion during colony initiation. We show that hyphal fusion in mature colonies between two incompatible strains leads to rapid cell death, while heterokaryon incompatibility is suppressed following CAT fusion during colony initiation. Heterokaryotic mycelia formed from CAT fusion survived with altered phenotypic traits.

## Results

### Labelling of *Colletotrichum* nuclei with red and green fluorescent proteins

In order to observe nuclear dynamics at different stages of CAT fusion during colony initiation and hyphal fusion in mature colonies, we used two plasmids to generate the strains in which nuclei were labelled with either GFP or RFP. Plasmid pMF357 [24] carries the *sgfp* gene fused to *N. crassa* histone H1, and contains the *hph* gene which confers hygromycin B resistance. Plasmid pGR02 carries the RFP gene for a dimeric version of dsRed (‘tdimer2(12)’; [25], [26]), fused to *F. graminearum* histone H4-2, and contains the *ble* gene which confers phleomycin resistance. Wild type strains LV115, LV51 and LV77 were transformed with these plasmids. The transformation efficiency of *C. lindemuthianum* varied between strains and plasmids used. The highest transformation efficiency (1.67 transformants µg^−1^ DNA) was obtained with LV115 with pMF357. Transformation efficiency was lower (0.2 transformants µg^−1^ DNA) when LV51 was transformed with pGR02. Despite numerous attempts, we were unable to transform LV77 with these plasmids. We refer to the LV115 transformant with H1-GFP as tFI01 and LV51 transformant with RFP-H4 as tFI04 (Table 1).

**Table 1:** *Colletotrichum lindemuthianum* strains used in this study.

Strain	Background	Origin or genotype*
LV115	NA	Patos de Minas (MG) –Brazil
LV51	NA	Lavras (MG) – Brazil
LV77	NA	Lavras (MG) – Brazil
tFI01	LV 115	*Pccg-1-hH1-sgfp; hph
tFI04	LV 51	*Pccg-1- tdimer2(12)- FghH4-2; ble

### Fusion between mature hyphae of incompatible strains results in rapid cell death

**Figure 1:**
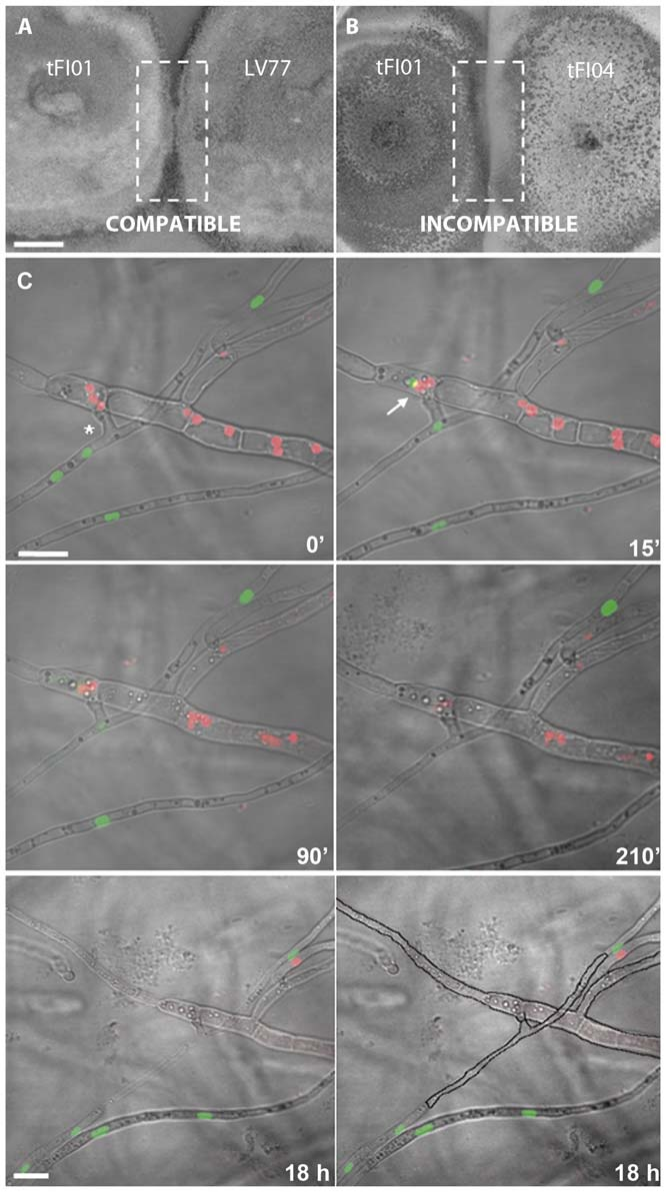
Heterokaryon compatibility/incompatibility responses between mature colonies. (A) Compatible response between tFI01 and LV77 on PDA medium plate. (B) Incompatible response between tFI01and tFI04 with formation of a ‘barrage’ in the region of contact (culture age: 35 days). (C) Time lapse imaging of nuclear dynamics and fate before and after vegetative hyphal fusion between tFI01 and tFI04 (incompatible combination) in which nuclei were differentially labelled. At time 0 min, hyphae of the two different strains have fused (asterisk). After fusion, nuclear migration occurred (15 min). The arrow indicates a single nucleus that has migrated between the fused hyphae. After 90 min, the green nuclei started to disappear. At 210 min, the fused hyphal compartments were mostly devoid of fluorescence, and nuclear fluorescence in adjacent compartments also decreased, suggesting that these hyphal regions were dying. These hyphal compartments were dead after 18 h. Black solid lines in the last panel outline shapes of the hyphae which have lost their cytoplasmic contents. Bars: 1 cm (A and B), 10 µm (C; note that the bar marker at 0′ relates to the 15′, 90′ and 210′ time points whilst the bar marker at 18 h refers to both panels shown at that time point).

Mature colonies of tFI01 and LV77 are vegetatively compatible because when confronted with each other they exhibited no macroscopic evidence of an incompatible response (Fig. 1A). Confrontation between mature colonies of tFI01 and tFI04, however, showed that they are vegetatively incompatible as they stopped growth and formed a ‘barrage’ in the region of contact (Fig. 1B). Thus, we used these two combinations to further investigate compatible and incompatible responses after hyphal fusion.

Zones of interaction between the two strains were analyzed by live-cell microscopy. Differential nuclear labelling facilitated a clear distinction of the origin for each hypha in the compatible and incompatible combinations. Following hyphal fusion between the incompatible pair (tFI01 and tFI04) nuclear migration occurred (Fig. 1C, 15 min) to form a heterokaryotic hyphal compartment. Approximately 90 min after hyphal fusion, nuclear fluorescence started to disappear in this compartment (Fig. 1C, 90 min), suggesting the initiation of cell death accompanied by degradation of the labelled nuclei. Nuclear fluorescence in adjacent hyphal compartments was decreased after 210 min. After 18 h, the fused hyphal compartment, and the three compartments adjacent to the fusion, were devoid of fluorescence and had lost their cytoplasmic contents. We observed five hyphal fusion events between the incompatible pair, and the fusions always resulted in a similar pattern of cell death in the fused hyphae.

**Figure 2:**
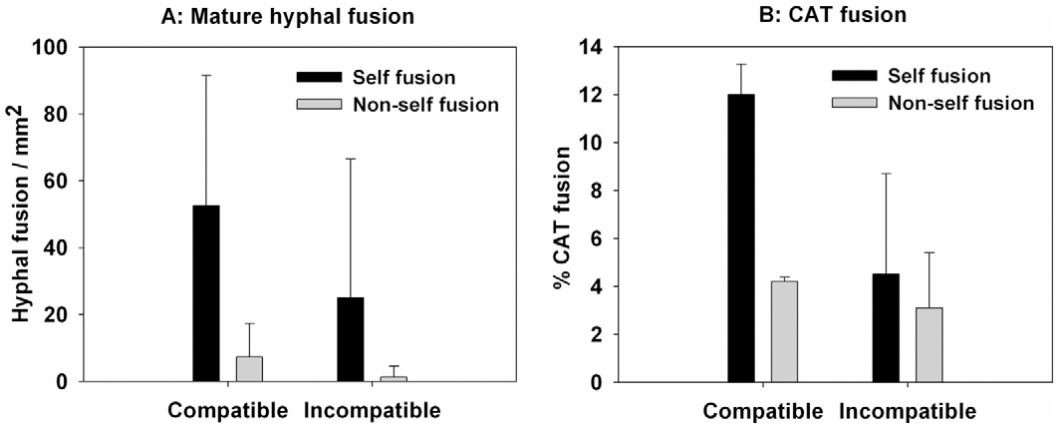
Quantification of hyphal fusion events. (A) The number of self and non-self vegetative hyphal fusion in the region of colony contact (see boxes in Fig. 1A and 1B) between compatible (tFI01and LV77) and incompatible combinations (tFI01and tFI04). The number of fusions per mm^2^ from a total of 10–12 combinations was counted. (B) Percentage of conidia involved in self- and non-self CAT fusions in the compatible and incompatible combinations after 48 h incubation of conidia harvested from 13 day-old cultures.

We then quantified the amount of fusion between hyphae of the same colony (self fusion) and between different colonies (non-self fusion) in the region of contact in both the compatible and incompatible combinations. Self fusion was relatively common in both the compatible (52 fusion per mm^2^) and incompatible combinations (25 fusions per mm^2^). Non-self fusions were rare, however, especially between the incompatible strains in which the amount of non-self fusion was less than 5% of all hyphal fusion observed (Fig. 2A). A χ^2^ test showed that there was no significant difference in the proportions of self and non-self hyphal fusions between the two combinations (*P* = 0.17). These results indicate that vegetative hyphal fusion between incompatible strains is restricted and leads to rapid cell death within a few hours.

The two incompatible strains tFI01 and tFI04 were expressing the *hph* and *ble* antibiotic resistance genes, respectively (Table 1). Pieces of mycelia from the interaction zone between the two incompatible strains, in which the occurrence of hyphal fusion had been microscopically confirmed, were removed from medium lacking antibiotics and plated on selection medium containing the two antibiotics. No mycelial growth was observed which further confirmed that vegetative incompatibility prevents the survival of heterokaryotic cells formed by hyphal fusion between mature colonies.

### A heterokaryon incompatibility response does not occur following CAT fusion

We next investigated the amount of CAT fusion in both compatible and incompatible combinations (Fig. 2B), and the fate of the resultant fused conidia or conidial germlings. Nuclei labelled with either GFP or RFP enabled us to distinguish between self and non-self fusions. Quantifying only fusion events between different strains (non-self fusion), the percentage of conidia involved in CAT fusion between compatible strains (tFI01 and LV 77) was 4.2±0.19% while that between incompatible strains (tFI01 and tFI04) was 3.1±2.3%. Non-self fusions between incompatible strains were commonly found (41% of all fusion events) and resulted in normal, healthy colonies during their early stage of development (<96 h, see below), in contrast to non-self fusions in mature colonies that resulted in cell death within 18 h following fusion (Fig. 1C). A χ^2^ test showed that there was no significant difference in the proportions of self and non-self CAT fusions between compatible and incompatible combinations (P = 0.57). However, a χ^2^ test comparing the proportions of self and non-self fusions between hyphae in mature colonies and CAT fusion in the incompatible combination showed that there was significant difference between them (*P*<0.01). This implies that incompatible hyphal fusion occurs more commonly during colony initiation than in mature colonies.

### Heterokaryotic cell formation by CAT fusion leads to the formation of mixed colour nuclei

**Figure 3:**
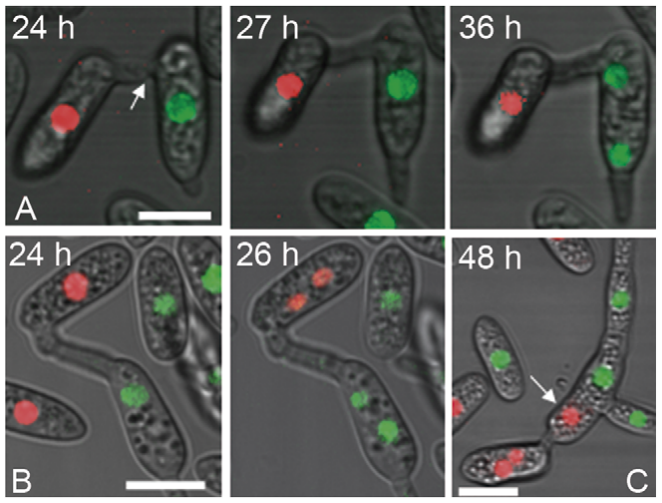
Time-lapse imaging of nuclear dynamics following CAT fusion between incompatible strains (tFI01 and tFI04). (A) CAT fusion was established (arrow at 24 h time point) and a germ tube started elongating from the base of the right conidium. Mitosis occurred in the right conidium between 27 and 36 h. (B) Following CAT fusion between uninucleate conidia (24 hrs), mitosis occurred after 2 h (26 h). (C) A nucleus (arrow) has migrated from one conidium to the other to form a heterokaryotic cell. Bar = 10 µm.

The fate of fused conidia or conidial germlings following CAT fusion between the incompatible strains (tFI01 and tFI04) was further investigated using confocal live-cell imaging. CAT fusion between two uninucleate conidia from incompatible strains was typically followed by mitosis (Fig. 3A, B), but not necessarily in both of the fused conidia (Fig. 3A). Following CAT fusion, fused conidia often formed germ tubes (Fig. 3A, 24 h) and underwent further CAT fusions (data not shown). In addition, nuclei were seen to migrate from one conidium to the other to form heterokaryons (Fig. 3C). Both green and red nuclei were capable of migrating through fused CATs (red, n = 6; green, n = 4).

**Figure 4:**
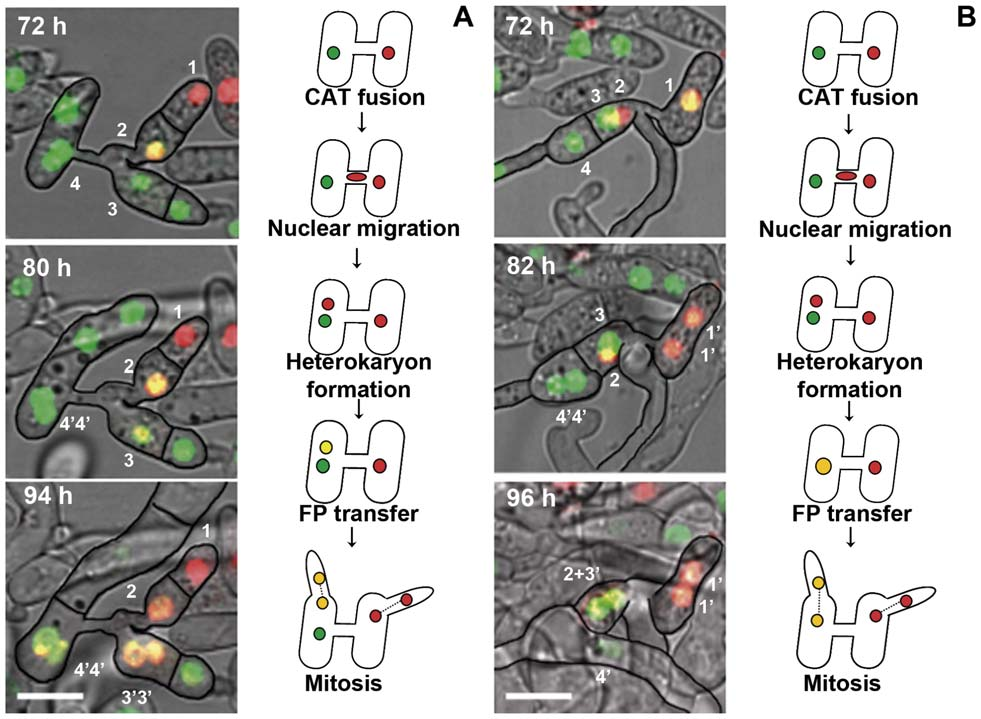
Two mechanisms by which yellow nuclei can be formed in heterokaryotic cells following CAT fusion. Conidia were incubated for 72 h (48 h incubation in water followed by 24 h incubation on PDA). The images of nuclei are shown as projections of z stacks of images with the green and red channels merged. (A) A green nucleus (nucleus 3) eventually turned greenish yellow (80 h), then to yellow when the nucleus underwent mitosis (94 h). Nucleus 4 also turned from green to greenish-yellow (94 h). (B) A red nucleus (nucleus 2) and a green nucleus (nucleus 3) became merged within the same focal plane, suggesting a nuclear fusion event (82 h). Bar = 10 µm.

Subsequent to heterokaryon formation following CAT fusion, the formation of yellow nuclei (which resulted from the co-existence of H1-GFP and RFP-H4 within single nuclei), was sometimes observed. After 72 h of incubation, yellow nuclei were observed in 27% of the heterokaryotic cells generated by CAT fusion (n = 60). There seemed to be two distinct mechanisms by which these yellow nuclei were formed: (1) by a fluorescent fusion protein encoded by one of the nuclei being transferred into a nucleus labelled with the other fluorescent protein; and (2) by the fusion of red and green fluorescing nuclei (Fig. 4). 70% of the nuclei seemed to be produced by mechanism (1) and 30% by mechanism (2) (n = 20). In Fig. 4A, a green nucleus (nucleus 3) eventually turned yellow, suggesting import of RFP-H4 into the H1-GFP-labelled nucleus (Fig. 4A). We also observed nuclei that had become yellow subsequently revert to either green or red (e.g. nucleus 2 in Fig. 4A, and nucleus 1 in Fig. 4B). This suggests that histone proteins can be shuttled back and forth between nuclei that share the same cytoplasm. The fusion of red and green nuclei (nuclei 2 and 3, Fig. 4B) resulted in a larger yellow nucleus being formed (compare images at 82 h and 96 h). The yellow nuclei were ∼1.5 times larger in volume than individual red/green nuclei, based on measurements from images collected in z stacks, and about the same size as nuclei just before mitosis (i.e. in the G2 phase of the cell cycle after DNA replication has taken place during the S phase; e.g. the nucleus 4 in the Fig. 4A). This result suggests that these yellow nuclei may be diploid. In either case, this finding suggests that nuclei in heterokaryotic cells are still active, and do not immediately undergo nuclear degradation that is accompanied by cell death following vegetative hyphal fusion between incompatible strains in mature colonies.

### Distribution of the two types of nuclei in the heterokaryotic colony is not uniform

**Figure 5:**
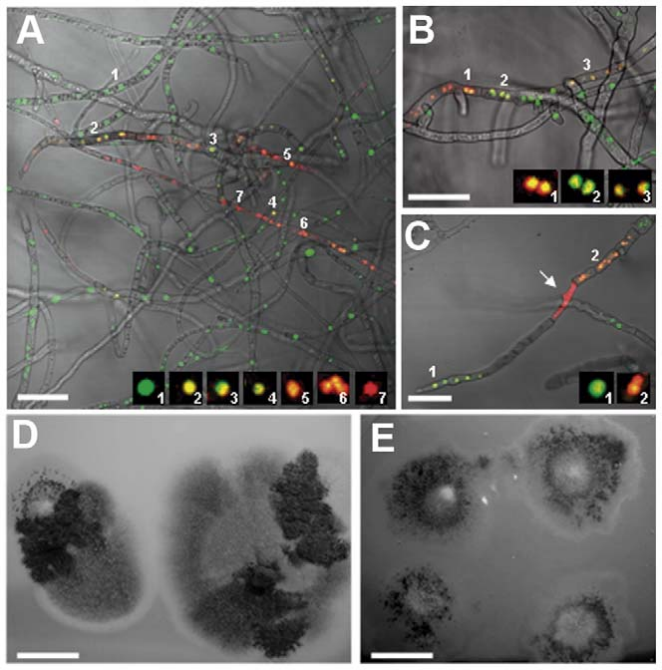
Heterokaryotic colony derived from a mixture of conidia from two incompatible strains that had undergone CAT fusion. (A) Nuclear distribution in the heterokaryotic colony was not uniform and green and red nuclei formed clusters. Insets show magnified images of nuclei with corresponding numbers in the lower magnification image. Yellow nuclei were present in the colony. The images of nuclei are shown as projections of z stacks of images with the green and red channels merged. (B) Another example of hyphae with nuclei of different colours. (C) A hypha undergoing apparent cell death, indicated by the red fluorescence dispersed throughout the cytoplasm (arrow) without any visible hyphal fusion nearby. Bars, 40 µm. (D) Subcultures of a heterokaryotic colony showing the formation of several distinct sectors. (E) Single conidial isolates exhibiting resistance to both Hyg and Phleo. Bar 15 mm.

Heterokaryotic colonies originating from CAT fusion were grown on double-selective medium for 7–15 days and imaged by confocal microscopy. Although hyphae with both green and red nuclei were observed (Fig. 5A), the distribution of labelled nuclei was not uniform in the colony (Fig. 5A, B). Green and red nuclei were generally separated from each other in different regions of an individual colony. Some hyphae underwent cell death without any detectable hyphal fusion having recently occurred in their vicinity (Fig. 5C), suggesting that the heterokaryon incompatibility response was delayed but still triggered in some heterokaryotic cells (i.e. a ‘leaky’ heterokaryon response). However, we also found yellow nuclei (Fig. 5A–C) in some regions in which cell death was not evident. Thus, some of the heterokaryotic cells formed by CAT fusion during colony initiation seemed to escape the incompatible response. The formation of dark grey or black mycelial sectors was observed in 15-day old heterokaryotic colonies that had been subcultured from the original heterokaryotic colonies and grown on both selective antibiotics (Fig. 5D). This suggested the presence of hyphae with different genotypes within the colony. This was never observed in the control experiments where mature hyphae of incompatible strains were fused.

### Emergence of hybrid or recombinant colonies from the heterokaryotic colony

The formation of heterokaryotic cells and mycelial sectors in colonies originating from CAT fusion suggested the emergence of hyphae with new traits. However, in order for these traits to have any physiological impact, the hyphae need to be capable of forming conidia to disseminate the new traits. To test this, we investigated whether the heterokaryotic colony forms conidia with both resistance markers. When conidia harvested from the control strains (tFI01 and tFI04) (lacking one of the antibiotics markers, Hyg or Phleo), were inoculated onto PDA containing both antibiotics, no colonies were formed. This clearly indicated that the antibiotics do not allow ‘background’ growth of any non-resistant colonies. In contrast, we observed colonies growing (Fig. 5E) on PDA containing both antibiotics when conidia from heterokaryotic colonies were used. There are at least two explanations for how conidia with both antibiotic markers are generated. One possibility is that heterokaryotic colonies form heterokaryotic, multinucleate conidia. The second possible explanation is that heterokaryon formation can sometimes lead to the fusion of haploid nuclei (suggested in Fig. 4), resulting in single nuclei that contain both markers. To differentiate between these two possibilities, we quantified the number of nuclei in conidia harvested from the parental strains (tFI01 and tFI04) and from heterokaryotic colonies. Virtually all conidia (99%±0.5) were uninucleate in the parental strains at 7 days. As cultures became older the percentage of anucleate and binucleate conidia increased (∼6% of conidia were anucleate and ∼1% binucleate in 16-day old cultures). In contrast, conidia from heterokaryotic colonies grown on selective media and stained with DAPI exhibited a high percentage of anucleate conidia (45.5%), while 53.7% of conidia were uninucleate and only 0.8% were binucleate in 20-day old cultures (n = 300). We then inoculated 100 µl of conidia from heterokaryotic colonies on each of four different growth media (M_3_S, M_3_S with Hyg, M_3_S with Phleo, and M_3_S with both antibiotics), and counted the number of colonies formed (Table 2). Since only 1.46% [0.8/(53.7+0.8)] of viable conidia could be binucleate, we would expect that the number of colonies formed on M_3_S containing the antibiotics to be much lower than that on M_3_S alone if heterokaryotic multinucleate conidia were responsible for the emergence of colonies expressing both antibiotic markers. However, the number of colonies on each medium was much higher and there was no statistically significant difference (*P* = 0.19) between the number of conidia formed on each medium (Table 2). This strongly supports the view that the colonies with the antibiotic markers were mostly derived from uninucleate conidia. These data suggest that, at least under these conditions, heterokaryon formation preferentially results in fusion of the two types of nuclei which can give rise to nuclei with new genotypes.

**Table 2:** Number of single conidia colonies originated from the heterokaryotic colonies on four different media (four replicates with three plates).

Selective Media	Average	SD
Medium without the two antibiotics	11.9	4.5
Medium with hygromycin;	9.5	4.3
Medium with phleomycin	14.9	5.4
Medium with both antibiotics (hygromycin and phleomycin)	7.7	3.9

### Putative recombinants or hybrids have different phenotypes to those of parental strains

We sought further confirmation of the idea that heterokaryon formation following CAT fusion can lead to emergence of strains with different genetic traits. We performed pathogenicity tests on differential bean cultivars and the Pérola cultivar (Table 3). Single conidia from the heterokaryotic colonies that had originated from CAT fusion were harvested and plated onto the medium containing both antibiotics. Conidia harvested from these colonies that were resistant to both Hyg and Phleo were used in pathogenicity tests with parental strains used as controls. The parental strain tFI01 showed pathogenicity that was identical to its parental strain LV115. However, pathogenicity of the tFI04 strain was classified as race 72 and different from that of its parental strain LV 51, which is classified as race 73. Four strains (R2, R3, R15 and R19) derived from the heterokaryotic colonies were tested for their pathogenicity and symptoms were only observed for R2, whose pathogenicity was classified as race 8. For the other three strains no symptoms were observed and they were classified as non-pathogenic. Interestingly, R2 did not cause disease symptoms in the Pérola cultivar used as a susceptible control, as opposed to the original strains which did cause disease symptoms (Table 3).

**Table 3:** Anthracnose disease susceptibility of different cultivars inoculated with the different strains of *C. lindemuthianum* used in this study and the race classification of these strains.

Cultivar*Strain	A	B	C	D	E	F	G	H	I	J	K	L	P	Race
LV115	+	−	−	−	−	−	+	−	−	−	−	−	+	65
LV 51	+	−	−	+	−	−	+	−	−	−	−	−	+	73
tFI01	+	−	−	−	−	−	+	−	−	−	−	−	+	65
tFI04	−	−	−	+	−	−	+	−	−	−	−	−	+	72
R2	−	−	−	+	−	−	−	−	−	−	−	−	−	8
R3	−	−	−	−	−	−	−	−	−	−	−	−	−	0
R15	−	−	−	−	−	−	−	−	−	−	−	−	−	0
R19	−	−	−	−	−	−	−	−	−	−	−	−	−	0

− Resistant (no symptoms); + Susceptible (with symptoms - score >3).

*Common bean differential cultivars used to classify *C. lindemuthianum* races followed by their binary value in brackets: A – Michelite (2^0^), B – Michigan Dark Red Kidney (2^1^), C – Perry Marrow (2^2^), D – Cornell 49242 (2^3^), E – Widusa (2^4^), F – Kaboon (2^5^), G – México222 (2^6^), H – PI 207262 (2^7^), I – TO (2^8^), J – TU (2^9^), K – AB 136 (2^10^), L- G 2333 (2^11^) and P – Pérola (susceptible control).

## Discussion

Accumulating evidence has highlighted the importance of the vegetative incompatibility response in preventing establishment of a heterokaryon between two incompatible fungal isolates [8]–[14]. Consistently, we have shown that hyphal fusion between mature hyphae of *C. lindemuthianum* that are vegetatively incompatible always results in rapid cell death of the fused cells. However, the remarkable finding that we have consistently demonstrated in this study is that heterokaryotic cells formed following CAT fusion between vegetatively incompatible strains survive and stable heterokaryons are maintained during the early stages of colony development following CAT fusion. This represents an escape from the normal incompatibility response resulting in cell death that we showed to occur in mature colonies of *C. lindemuthianum*. Thus, our results indicate that vegetative incompatibility can be suppressed during colony initiation and that CAT fusion may facilitate horizontal gene/chromosome transfer and non-meiotic recombination of genetic information from two incompatible strains to promote expansion of genetic diversity. Suppression of the vegetative incompatibility response following CAT fusion in *C. lindemuthianum* has been previously proposed [16], [20], [27], [28] but strong experimental data to support this proposal have not been obtained until the present study. We directly monitored CAT fusion between incompatible strains and the resultant fused conidia survived for at least 30 h without undergoing cell death. In contrast, we found that nuclei and cell death occurred within ∼3 h following fusion between mature hyphae of incompatible strains of *C. lindemuthianum*. These findings challenge the previous idea that genetic exchange between two incompatible strains will be invariably prevented following vegetative fusion [8], [9].

Time-lapse imaging of heterokaryotic cells resulting from CAT fusion revealed the formation of yellow fluorescing nuclei containing both green and red fluorescently labelled histones. The yellow nuclei seemed to be formed in two different ways (Fig. 4), either by the incorporation of both fluorescent proteins in one nucleus, or by the fusion of green and red nuclei. Yellow nuclei were never observed to form in the mature colony following vegetative hyphal fusion between incompatible strains. Some hyphae within mature heterokaryotic colonies that had formed by CAT fusion did undergo cell death without any apparent hyphal fusion event in their vicinity. This may represent a ‘leaky’ heterokaryon response. However, we did find that all three types of nuclei (i.e. green, red and yellow fluorescing nuclei) still co-existed in a single colony, suggesting that at least some heterokaryotic hyphae in mature colonies can escape cell death.

We also obtained evidence of non-meiotic genetic recombination occurring following CAT fusion because we were able to grow colonies exhibiting resistance against both Hyg and Phleo from uninucleate conidia harvested from heterokaryotic colonies. As the parental strains did not form any colonies on plates with both antibiotics, these putative recombinant or hybrid colonies are not merely background survivors and are thus likely to be carrying the both resistance genes. The ratio of uninucleate to binucleate conidia and the number of colonies formed on plates with or without the antibiotics provided strong evidence that the colonies resistant to both antibiotics arose from uninucleate conidia. This further supports the occurrence of fusion between two nuclei of different genotypes and the resultant formation of diploid nuclei in the heterokaryotic colonies. Since our persistent attempts to determine the ploidy of conidia by flow cytometry were unsuccessful (data not shown), it is not yet clear whether the heterokaryotic colonies form diploid, haploid or aneuploid nuclei. It has been suggested that if a diploid nucleus is formed during the vegetative stage, somatic crossing over or non-disjunction can sometimes occur to result in haploidization, with resultant conidia either having diploid nuclei homozygous for certain genes or having haploid nuclei with new combinations of genes [29]. Previous attempts to demonstrate a parasexual cycle in *C. lindemuthianum* were unsuccessful in convincingly showing the presence of diploid nuclei [7], [30], [31], and this may be because diploid states are transient and followed by rapid aneuploidization during mitosis. In either case, our data imply that new genotypes could arise from two incompatible strains via CAT fusion.

Disappointingly, despite numerous attempts, we have so far been able to detect only the *phleo* marker, but not the *hph* marker, in the genomes of putative recombinant or hybrid colonies by Southern analyses (data not shown). As mentioned above, the putative recombinant/hybrid colonies do not seem to be merely background survivors and are thus likely carrying at least a small amount of the hygromycin phosphotransferase. This apparent contradiction might be explained by observations from *F. oxysporum* where only a certain lineage-specific (LS) chromosome can be stably maintained after horizontal gene transfer [32]. Similarly, an *hph* selectable marker could be only transferred between two incompatible biotypes of *C. gloesporioides* when integrated in a supernumerary chromosome (2-Mb) but not when integrated in other chromosomes [22]. Very recently, Manners and He [23] successfully generated heterokaryons from a mixture of conidia of the same two incompatible biotypes, and found a biased copy number of the two genomes in the heterokaryotic mycelium. Based on this result, these authors suggested that a particular nuclear balance is selected to provide some heterokaryon compatibility. Since the vectors were randomly inserted in our study, the chromosome in which *hph* has been integrated may not be maintained stably after hyphal fusion. It is possible that the diploid nuclei lose some of their chromosomes, and that the *hph*-marked chromosome is maintained at levels that were below the detection limit for the Southern assay. Nevertheless, pathogenicity tests clearly demonstrated that the putative recombinants or hybrids have different pathogenic traits from the parental strains. Two strains used as parental strains showed differences for two virulence factors (see Table 3). The strain tFI01, race 65, is pathogenic to Michelite (2^0^) and México 222 (2^6^) cultivars, and tFI04, race 72, is pathogenic to México 222 (2^6^) and Cornell 49242 (2^3^). The pathogenicity test showed that we obtained one strain (R2) classified as race 8 that was only pathogenic to the Cornell 49242 cultivar (2^3^). The virulence factor to México 222 (2^6^) cultivar was lost in all the recombinants. Thus, heterokaryon formation as a consequence of CAT fusion at least leads to the generation of new strains with distinct phenotypes, which may be achieved by the generation of new genotypes.

Although the importance of non-meiotic recombination for generating genetic variability has long been recognized, most studies have not investigated mechanisms by which heterokaryotic cells are formed prior to recombination. With genome sequencing of several organisms, it became possible to identify common sequences in different species, which suggests horizontal gene transfer between their ancestors [6], [32], [33], [34] or otherwise distantly related fungi [35]. For example, horizontal gene and chromosome transfer of host-specific factors between otherwise distant and genetically isolated lineages of *F. oxysporum* may thus explain the apparent polyphyletic origins of host specialization and rapid emergence of new pathogenic lineages [32]. The role of CAT fusion in this process has not been shown although CAT fusion has been described during the early stages of infection by *F. oxysporum* [36].

CAT fusion may account for horizontal gene and chromosome transfer between otherwise incompatible strains [6], [32] and also be important for the occurrence of other chromosome polymorphisms such as supernumerary chromosomes described in several plant pathogenic fungi [22], [33], [34], [37], [38], [39] including *C. lindemuthianum* [40]. The horizontal transfer of chromosomes responsible for pathogenicity has been recently described in *Fusarium oxysporum* [32]. CAT fusion may also be important in the evolution of interspecies hybrids [20] and in giving rise to new plant pathogens [35], [41], [42]. Research on the underlying mechanisms mediating horizontal gene or chromosome transfer, and particularly the role of CAT fusion in these processes, should improve our understanding of how genetic diversity of filamentous fungi is achieved, and should provide new strategies for controlling emerging plant pathogens.

## Materials and Methods

### Strains and culture conditions

*Colletotrichum lindemuthianum* strains used in this study were derived from the culture collection of the Department of Biology, Universidade Federal de Lavras (Table 1), Brazil. All strains were grown and maintained on Potato Dextrose Agar (PDA) or M_3_S medium [43]. To stimulate sporulation, autoclaved French bean pods with 2% water agar were inoculated with the different strains [44].

### Plasmids

Plasmid pGR02 was constructed by digesting pLC11, which contains tdimerRed, (RFP; [26]) fused to the *histone H4-2* gene (FGSG_05491.3) from *Fusarium graminearum* PH1 under the control of the *Neurospora crassa ccg-1* promoter, with ApaI and XbaI. The purified restriction fragment was ligated into ApaI- and XbaI-digested pBC-phleo [45], which contains the *ble* gene under the control of the *Aspergillus nidulans gpdA* promoter to confer phleomycin (Phleo) resistance. Plasmid pMF357 carries the *histone H1-sgfp* fusion gene under the control of the *Neurospora ccg-1* promoter, and the *hph* gene to confer hygromycin (Hyg) resistance [24]. Cloning and preparation of plasmids were performed using standard laboratory protocols [46]. All plasmids were maintained in the *Escherichia coli* strain DH5α and purified with Qiagen DNA miniprep columns (Qiagen, Hilden, Germany).

### Genetic transformations

Conidia were harvested from 10–15 days-old cultures of the *C. lindemuthianum* strains (Table 1), which were washed twice in water, and used to inoculate 100 ml M_3_S medium [43]. Protoplasts were obtained [47] and transformation was carried out as previously described [44]. Briefly, protoplasts in STC (1.2 M sorbitol, 10 mM Tris-HCl and 50 mM CaCl_2_, pH 7.5) at a concentration of 10^7^ protoplasts in 100 µl were combined with 3–5 µg of plasmid DNA (either pMF357 or pGR02) in a 50-ml polypropylene centrifuge tube. The mixture was incubated on ice for 20 min, 1 ml of PEG (40% PEG 3,350, 0.6 M KCl, 50 mM CaCl_2_ and 50 mM Tris-HCl, pH 8.0) was added and the mixture incubated at room temperature for 20 min. Regeneration agar (40 ml; 1 M sucrose, 1.25% casein hydrolysate, 1.25% yeast extract, 1.0% agar, pH 8.0) was added. Accurate pH adjustment was important for selection on Phleo containing medium. Transformants were selected based on resistance to antibiotics (50 µg ml^−1^ Hyg or 25 µg ml^−1^ Phleo). After transformation, Petri dishes were incubated at 22°C, and after 7 days Hyg- or Phleo-resistant colonies were transferred to PDA containing the same amounts of antibiotics. Conidia from these transformants were spread onto PDA containing the respective antibiotic and after 48 h, single Hyg- or Phleo-resistant germlings were recovered from each plate and transferred to PDA+Hyg or PDA+Phleo. Single conidia that expressed H1-GFP or RFP-H4 were selected under a fluorescence stereomicroscope (Nikon SMZ1500, Nikon, Kingston-Upon-Thames, UK) with filter sets appropriate for dsRED (excitation 545/30 nm, emission 620/60 nm) and GFP (excitation 460/50 nm, emission 500/30 nm). The strain that carries pMF357 and expresses H1-GFP was designated tFI01, while the strain that carries pGR02 and expresses RFP-H4 was designated tFI04.

### Light and confocal microscopy

We used an inverted Nikon TE2000E microscope equipped with 40× (1.3 N.A.) oil immersion and 60× (1.2 N.A.) water immersion plan apo objectives for routine light microscopy. Confocal laser scanning microscopy was performed with a BioRad Radiance 2100 system equipped with blue diode and argon ion lasers mounted on a Nikon TE 2000 U Eclipse inverted microscope. GFP and RFP were imaged simultaneously by exciting with the 488 and 543 nm laser lines and fluorescence detection was at 500/30 nm (for GFP) and >560 nm (for RFP). Confocal images were captured with the Lasersharp software (version 5.1; BioRad). Images were transferred into Imaris (version 4.1), Image J (version 1.42q) or Paintshop Pro software (version 7.0) for further processing.

### Heterokaryon compatibility assays

Heterokaryon compatibility between strains was investigated by a conventional confrontation assay (10 replicates), in which blocks of agar containing the mycelium were placed 4–5 cm away from each other on PDA in Petri dishes, and the presence of hyphae in the contact area was assessed macroscopically. To better observe hyphal fusion between colonies, a sheet of sterile cellophane was placed on PDA between the two colonies, and after mycelial growth had taken place the cellophane along with mycelia was mounted on a cover glass and imaged by confocal microscopy (see previous section). The number of vegetative hyphal fusion events in five 195×195 µm squares was quantified (n = 10–12).

### Observations of CAT fusion

Conidia were collected from 13–17-day-old cultures, suspended in water, and their concentration adjusted to 10^6^ spores per ml. For CAT fusion, equal proportions of conidial suspensions from the tFI01 and either tFI04 or LV77 strains were mixed and incubated in the dark at 22°C for 48 h. For imaging samples and for the quantification of CAT fusion, 200 µl drops of conidial suspension were place in an eight-well slide culture chamber (Nalge Nunc International, Rochester, NY). For time lapse imaging of nuclear dynamics in conidia and conidial germlings, we used the inverted agar block method [48] with minor modifications. To facilitate clear observations of nuclear dynamics, the conidial mixture was first incubated for 48 h at 22°C to allow CAT fusion to occur, and then the fused conidial germlings were diluted tenfold with distilled water and plated on solid PDA medium, followed by further 24 h incubation at 22°C. Confocal live-cell imaging was performed over a 24 h period to monitor nuclear organization and dynamics at the different stages of CAT fusion. The conidial mixture, maintained in water for 48 h to allow CAT fusion [44], was plated on selective medium (PDA+Hyg+Phleo) to evaluate heterokaryon colony formation over a period of 7–15 days.

### Selection of putative recombinants/hybrids

Conidia were collected from the four different heterokaryotic colonies grown on PDA plates containing both Hyg and Phleo. The conidia were suspended in water and 100 µl of diluted conidial suspensions each containing 200 conidia.ml^−1^ were plated on: (i) medium without antibiotics; (ii) medium with Hyg; (iii) medium with Phleo; (iv) medium with both antibiotics (Hyg and Phleo). After 7–10 days, we counted colonies that had grown on each plate. The putative recombinants/hybrids that had grown on double-selective media (PDA+Hyg+Phleo) were purified by isolation of single conidia. To count nuclei, conidia from heterokaryotic colonies were suspended in PBS at a concentration of 10^6^ conidia ml^−1^ and their nuclei stained with 300 nM DAPI (4′,6-diamidino-2-fenilindole, Invitrogen) from a 30 mM stock in ethanol.

### Pathogenicity assays

Transformants tFI01 and tFI04 and some of the putative recombinants or hybrids were used to inoculate twelve different bean cultivars [49]; the “Pérola” bean cultivar was used as a susceptible control. Eight seeds of each cultivar were sown in 128-well polystyrene trays with Plantmax® planting mix (Eucatex, Paulina, SP, Brazil). After the emergence of the primary leaves, each tray with seedlings was sprayed with 200 ml of conidial suspensions (1.2×10^6^ spores ml^−1^) that were harvested from 15–20 day-old cultures, and the trays were placed in a moist chamber at 20±2°C with a photoperiod of 12 h dark/12 h light. Seedlings were incubated for 10 days, after which symptoms of infected plants were evaluated on a scale from 1 to 9, in which scores of 1–3 represent resistant plants and scores 4–9 represent susceptible plants [50]. The Habgood [51] binary system was employed for the race identification.

### Statistical analysis

Proportions of self and non-self fusion were compared between compatible and incompatible combinations, involving hyphal fusion in mature colonies and CAT fusion, using the Chi-square (χ^2^) test. The number of colonies originating from conidia derived from heterokaryotic colonies grown on plates with different combinations of antibiotics was compared by an analysis of variance (ANOVA) using the statistical program MSTAT-C 1.0 (Freed, R.; MI, USA).
